# Balancing Open-Mindedness and Sound-Mindedness: A Physician’s Perspective

**DOI:** 10.7759/cureus.51948

**Published:** 2024-01-09

**Authors:** Kalimullah Jan

**Affiliations:** 1 Neurology, Westchester Medical Center, New York Medical College, Valhalla, USA

**Keywords:** medical school education, health care outcomes, logic, medical decision making, open-mindedness

## Abstract

Open-mindedness in the medical decision-making process is fundamental as it aids in averting mistakes, yet it can also breed ambiguity if it’s too excessive. On the other hand, sound-mindedness, which is a balanced method that employs logic and evidence in problem-solving, could be the preferred approach. Both these traits have their limitations, yet they can supplement each other in various clinical contexts. Therefore, it’s crucial for medical professionals to wisely cultivate and uphold both these traits.

## Editorial

The value of open-mindedness, a quality revered since the era of Socrates, is recognized as a crucial element for learning and understanding in the eyes of today's learning theorists [1]. In my journey as a medical professional, working in a variety of settings and health systems, catering to diverse patient demographics in four different countries, one constant theme I have acknowledged is the pivotal role of harboring an open mind. This mindset is a key factor in avoiding rushed conclusions, which can often result in diagnostic and treatment errors, and consequently, suboptimal patient outcomes. Unfortunately, real-world data shows that medical errors still occur, largely due to cognitive factors, and are a significant contributor to mortality and morbidity [2].

While keeping an open mind is essential in medicine, it’s crucial to understand that each clinical problem comes with a multitude of theoretical possibilities. One could argue that "too much" open-mindedness can sometimes be counterproductive, especially when it’s naive or indiscriminate, leading to uncertainty and vulnerability to misinformation and emotionally driven literature rather than logic and factual data.

Therefore, a balanced approach, or sound-mindedness, is often ideal. For example, a diabetic patient on insulin had a brief episode of slurred speech and mild confusion in the setting of low blood glucose of 40 mg /dl. The symptoms quickly corrected with the administration of 50 ml of 50% dextrose in the emergency room. A team member suggested a transient ischemic attack (TIA) workup in the name of open-mindedness. This was unnecessary as there were no other TIA indicators and the patient met Whipple’s triad criteria for hypoglycemia. Unwarranted open-mindedness in such situations can lead to unneeded and potentially harmful investigations or interventions. The team member acknowledged that the TIA workup suggestion was driven by fear of missing a diagnosis and potential litigation, highlighting a drawback of the current medical culture.

Sound-mindedness, the ability to apply logic, evidence, and consistency to information processing and problem-solving, can help avoid errors and biases that potentially impair clinical reasoning. It can help distinguish between correlation and causation, recognize confounding variables and placebo effects, and test hypotheses using rigorous methods. However, sound-mindedness can also be detrimental when it becomes rigid or narrow-minded, leading to overconfidence or complacency. It can also be influenced by social and emotional factors such as peer pressure and groupthink, compromising objectivity.

In conclusion, both sound-mindedness and open-mindedness are valuable cognitive traits in medicine, each with its drawbacks and limitations. They can complement each other depending on the clinical context. Therefore, medical professionals should strive to develop and maintain both traits, using them wisely.

## References

[1] Lord M (2015). Group learning capacity: the roles of open-mindedness and shared vision. Front Psychol.

[2] Saposnik G, Redelmeier D, Ruff CC, Tobler PN (2016). Cognitive biases associated with medical decisions: a systematic review. BMC Med Inform Decis Mak.

